# Pharmacogenomic Drug–Target Network Analysis Reveals Similarity Profiles Among FDA–Approved Cancer Drugs

**DOI:** 10.3390/pharmaceutics17111421

**Published:** 2025-11-03

**Authors:** Alberto Berral-González, Monica M. Arroyo, Diego Alonso-López, María Jesús Rivas-López, José Manuel Sánchez-Santos, Javier De Las Rivas

**Affiliations:** 1 Cancer Research Center (CiC-IBMCC, CSIC/USAL), Consejo Superior de Investigaciones Científicas (CSIC)/University of Salamanca (USAL), & Instituto de Investigación Biomédica de Salamanca (IBSAL), 37007 Salamanca, Spain; aberralgonzalez@usal.es (A.B.-G.); jose@usal.es (J.M.S.-S.); 2 Chemistry Department, Pontifical Catholic University of Puerto Rico (PCUPR), Ponce 00717, Puerto Rico; monica_arroyo@pucpr.edu; 3 Bioinformatics Unit, Cancer Research Center (CiC-IBMCC, CSIC/USAL), Consejo Superior de Investigaciones Científicas (CSIC)/University of Salamanca (USAL), 37007 Salamanca, Spain; diego.alonso@usal.es; 4 Department of Statistics, University of Salamanca (USAL), 37008 Salamanca, Spain; chusrl@usal.es; 5 Instituto de Física Fundamental y Matemáticas (IUFFyM), University of Salamanca (USAL), 37008 Salamanca, Spain

**Keywords:** cancer, drug, drug–target, anticancer drug, protein-drug network, oncological pharmacology, pharmacogenomics, interactomics, bioinformatics

## Abstract

**Background**: Defining specific molecular targets for cancer therapeutics remains a significant challenge in oncology. Many Food and Drug Administration (FDA)-approved anticancer drugs have incomplete target profiles, which limits our understanding of their mechanisms of action and opportunities for drug application. In this context, this study aimed to establish novel, biologically meaningful relationships between anticancer drugs and protein-coding genes. **Methods**: We developed a pharmacogenomic method that integrates transcriptomic data with drug activity data from the NCI-60 cancer cell line panel to study the interactions between 124 FDA-approved anticancer drugs and 399 cancer-related genes. Through this analysis, we identified gene–drug relationships and created a bipartite interaction network. To evaluate drug similarity, we developed a new index called the B-index. This novel similarity coefficient measures the association between two drugs based on their shared gene targets in the network. The index calculates the intersection of two sets of drug targets while considering the relative proportion of targets exhibited by each drug. For an independent assessment, we compared this network-based similarity with the chemical structural similarity of the drugs, computed based on two structural coefficients: Maximum Common Substructure and Tanimoto. **Results**: The study identified 1304 statistically significant drug–gene relationships, providing a large-scale network of pharmacogenomic interactions. Clustering analysis of the network, based on the B-index, grouped drugs with common targets together. This grouping was consistent with well-established drug classes and structural characteristics. Well-established drug pairs, such as cytarabine–gemcitabine or afatinib–neratinib, exhibited high B-index and structural similarity values, validating the methodology. Several novel gene associations were discovered, yielding testable hypotheses for mechanism-based repurposing. **Conclusions**: This work presents a comprehensive, network-based strategy for elucidating cancer drug targets by combining gene expression and drug activity profiles. Additionally, the B-index provides an alternative to conventional chemical similarity metrics, which can facilitate the identification of new therapeutic relationships and inform new drug applications and repositioning. These findings pave the way for the proposal of novel oncology drug targets.

## 1. Introduction

The cancer therapeutic arena has expanded substantially, with many therapeutic agents receiving approval from the world’s premier drug-regulating bodies, including the U.S. Food and Drug Administration (FDA) and the European Medicines Agency (EMA), over the last decades [1]. Despite this therapeutic expansion, the molecular mechanisms of action of many anticancer drugs remain only partly understood, particularly with respect to the precise protein targets, and the corresponding protein-coding genes, of novel chemical compounds produced by the pharmaceutical industry. This gap in knowledge limits the ability to predict resistance, optimize dosing, identify biomarkers of response, and repurpose existing drugs for new indications.

The identification of molecular targets for specific drugs is a critical step to improving disease therapy. The generation of maps of anticancer drugs and their target genes began several years ago thanks to the production of many omic data about the activity of the drugs correlated with the activity of the human genes [2]. Pharmacogenomic approaches have been developed as high-throughput methods for linking pharmacological response and gene expression profiles in genetically heterogeneous cellular models. The NCI-60 cancer cell line panel, available via the CellMiner portal, offers merged drug sensitivity and gene expression data for an extensive collection of FDA-approved drugs [3]. The Catalogue Of Somatic Mutations In Cancer (COSMIC) houses the Cancer Gene Census (CGC), which is a curated list of genes with proven roles in human cancer [4,5]. These resources provide information about cancer-associated genes and enable researchers to investigate possible drug–gene interactions using cancer cell lines, thereby bringing together molecular oncology and pharmacology. However, a deeper understanding of drug–protein interactions at a molecular level is essential for advancing causal discovery in cancer and for developing novel anticancer therapies based on identifying new molecular targets. In this respect, pharmacogenomic profiling has been employed to link gene expression signatures to drug response phenotypes in the NCI-60 and other cell line panels [6], although functional similarities beyond overlapping structural motifs remain under-explored.

In this study, we aim to systematically evaluate FDA-approved anticancer drugs using pharmacogenomic data, which provides information on drug sensitivity and full gene expression across 60 different cancer cell lines [3]. In this way, following a similar approach to that of Reinhold et al. [6], we implemented a robust correlation analysis between the drug sensitivity profiles and the gene expression profiles to identify putative associations between anticancer drugs and human protein-coding genes. Additionally, we also provide a novel approach to measuring the similarity between two drugs based on the gene targets that they share. To accomplish this, we need to apply an index that calculates the intersection of two sets of gene targets corresponding to the two query drugs. Measuring drug similarity based on shared gene targets requires suitable similarity measures. Classic measures, such as the Jaccard index, penalize similarity for asymmetrically sized gene sets (i.e., when the two groups have very different size), which is a frequent scenario in pharmacogenomic data. To address this limitation, we formulated and applied a new index, called the B-index (i.e., Berral-index), that adjusts for set size by averaging the cardinality of the gene sets assigned to each drug pair. Thus, the method takes into consideration the relative proportion of targets that each drug exhibits. The method prioritizes meaningful overlaps in small sets, revealing biologically relevant similarities that classic measures may obscure [7]. We confirm these gene-based connections through cross-validation using structural similarity measures, such as the Tanimoto coefficient and Maximum Common Substructure (MCS) coefficient [8,9]. This integrative approach identifies functionally and structurally concordant drug pairs, offering a new paradigm for discovering therapeutic possibilities in cancer treatment, and uncovering meaningful drug–gene relationships. Overall, these pharmacogenomic networks of drugs and genes will support current efforts to discover new cancer biomarkers and repurpose anticancer drugs.

## 2. Materials and Methods

### 2.1. Data Resources and Integration

To characterize drug–gene interactions in cancer, we integrated curated transcriptomic and pharmacologic activity data with annotated gene–disease associations. We obtained full transcriptomic profiles of protein-coding genes from the NCI-60 cancer cell lines using the CellMiner resource (v2.10, updated 2 March 2023) [6,10]. This pharmacogenomics database comprises matched expression and activity data for over 24,850 compounds, including over 170 FDA-approved oncology agents [3,11].

Gene expression was normalized and converted to average z-scores across five transcriptomic platforms (Affymetrix HG-U95, HG-U133, HG-U133 Plus 2.0, Human Exon 1.0 ST, and Agilent Whole Human Genome arrays), as described by Gmeiner et al. [12]. Drug activity across the 60 cell lines was measured using the sulforhodamine B assay and expressed as −log_10_(GI_50_) values. Both datasets were further standardized and averaged as z-scores to enable cross-drug comparisons [3,6].

Cancer genes were obtained from the Cancer Gene Census (CGC) release 98, a manually curated subset of the COSMIC database (updated 23 May 2023) [4,5]. Of 738 available CGC genes, 399 intersected with CellMiner profiles and met our filtering criteria for analysis. Drugs were ranked by FDA approval status based on the Developmental Therapeutics Program (DTP) oncology drug set (https://dtp.cancer.gov/organization/dscb/obtaining/available_plates.htm) (accessed on 5 July 2023). Compounds were designated by their NSC (National Service Center) number. DrugBank version 5.1.12 (released on 14 March 2024) was used to obtain empirical biomolecular target information and confirm the gene–drug relations identified in our analysis. This integrated approach enabled confident identification of transcriptomic–pharmacologic relationships with potential implications for repurposing, mechanism elucidation, and drug classification.

### 2.2. Statistical Analysis: Robust Calculation of Gene–Drug Correlations

Statistical analysis was conducted using the R programming environment (R Project for Statistical Computing, https://www.r-project.org/, version R-4.3.1) and the Hmisc package (version 5.2-4), following CellMiner methodological guidelines [6]. Gene expression and drug activity data for the NCI-60 panel were normalized and z-scored to ensure consistency across compounds and cell lines. Pearson and Spearman correlation coefficients were then calculated between each FDA-approved anticancer drug and each cancer-relevant gene across all 60 cell lines.

To ensure both statistical rigor and biological relevance, a multistep filtering approach was applied. Compounds with weak or anomalous activity profiles were first removed, as previously described [13]. Multiple testing correction was performed using the Holm method, which effectively controls the family-wise error rate and is well-suited for large-scale correlation analysis on dependent data, as commonly seen in pharmacogenomic datasets. Following CellMiner conventions, only correlations with Spearman’s ρ or Pearson’s r above 0.334 were retained—thresholds representing moderate-to-strong associations, chosen to emphasize biological signal while minimizing false discoveries [6].

FDA approval status was determined using the Developmental Therapeutics Program (DTP) oncology drug set (https://dtp.cancer.gov/organization/dscb/obtaining/available_plates.htm) (accessed on 5 July 2023), with each compound mapped via its NSC (National Service Center) identifier. Two drug sets were created: (i) FDA-approved anticancer agents and (ii) investigational or unapproved compounds, with only the former included in the final gene–drug network analysis. This rigorous filtering strategy yielded a high-confidence interaction set grounded in statistical significance, effect size, and pharmacological relevance across the NCI-60 panel, forming the foundation for all downstream network analyses.

### 2.3. Calculation of a Similarity Score Based on the Number of Shared Gene Targets

To assess drug similarity based on shared gene targets, we developed a novel similarity measure called the B-index (Berral index). Unlike conventional similarity coefficients such as the Jaccard index, which can be penalized by uneven set sizes [7,14], the B-index emphasizes overlaps that are particularly meaningful in small gene sets—a common occurrence in pharmacogenomic data.

For two non-empty sets x and y, the B-index is defined as:

$$B_{index}\left(x,y\right)=B(x,y)=\frac{1}{2}\cdot \left|x\cap y\right|\cdot \left(\frac{1}{\left|x\right|}+\frac{1}{\left|y\right|}\right)$$

This index ranges from 0 (no shared elements) to 1 (complete overlap) and satisfies the mathematical properties of a similarity function: non-negativity, symmetry, and the property that B(x,y) ≤ B(x,x) with equality if and only if x = y.

The B-index’s key advantage lies in its behavior with sets (i.e., groups of elements) of different sizes. When comparing a small set to a larger one, the B-index gives greater weight to their intersection than other traditional overlap measures, such as the Jaccard or Dice indices. For instance, if set x has 4 elements and set y has 40 elements and they share 2 elements, the B-index provides a similarity of 0.275, whereas the Jaccard index provides a similarity of 0.045. Thus, the B-index recognizes the similarity between these two groups as more significant than a conventional similarity index, such as the Jaccard index, because it weights overlapping elements proportionally to each set’s size. One shared element from a small set is statistically more likely to be selected (1/4 = 0.25 in set x in the example) than the same element from a large set (1/40 = 0.025 in set y).

The mathematical relationship between similarity indices follows the ordering: Russell-Rao [15] ≤ Jaccard [14,16] ≤ Sørensen-Dice [17,18] ≤ Berral, with the B-index (Berral) consistently providing the highest similarity scores when meaningful overlaps occur in small gene sets. Detailed mathematical proofs demonstrating that the B-index satisfies the formal requirements of a similarity function, derivations of the ordering relationships between similarity indices, and additional worked examples illustrating the index’s behavior with different set sizes are provided in File S1 (Mathematical foundations and proofs for the B-index).

### 2.4. B-Index Calculation and Clustering Analysis

To assess pharmacogenomic similarity among anticancer agents, we applied the B-index to estimate the gene set overlap between FDA-approved cancer drugs. As indicated before, the B-index accounts for the sizes of both gene sets independently, assigning greater weight to overlaps in smaller, more informative sets.

We generated 124 gene sets by identifying genes highly correlated with drug activity in the NCI-60 cell lines, as described above. Pairwise B-index values were computed to form a 124 × 124 symmetric similarity matrix, where values range from 0 (no overlap) to 1 (identical sets), with diagonal entries indicating self-similarity.

As the B-index is a similarity measure rather than a distance, clustering was performed after converting the similarity values into distance measures. For this, we employed R version 4.3.1, invoking the function *hclust* (version 3.6.2) with agglomeration method Ward.D2 to obtain maximum within-cluster homogeneity and a robust classification of drugs based on their shared gene associations. Fifteen drug clusters were identified as the optimal based on the highest relative loss of inertia criterion at varying clustering thresholds, as originally proposed by the HCPC function in the *FactoMineR* package (version 2.12). Finally, the B-index matrix, transformed into a distance matrix, was visualized as a heatmap using the *gplots* package. The resulting heatmap includes a color scale ranging from white (no similarity) to red/blue (high similarity), and a dendrogram showing the clustering of the anticancer drugs.

### 2.5. Drug Structural Analysis

To complement the pharmacogenomic similarity analysis and provide independent, orthogonal validation of the clustering of drugs (derived from the B-index), a structural comparison of 124 FDA-approved anticancer drugs was conducted using cheminformatics methods based on molecular fingerprints. These methods include the Tanimoto coefficient [16] and the calculation of the Maximum Common Substructure (MCS) [7] between each pair of drugs.

Drug identifiers (NSC codes, SMILES) were processed with ChemMine Tools (https://chemminetools.ucr.edu/) (accessed on 1 July 2023) to generate molecular descriptors and Structure Data Files (SDFs) [8]. Open Babel chemical toolbox (version 3.1.0) was used to compute fingerprints and physicochemical properties for standardized input [19]. Pairwise structural similarity was assessed using the *fmcsR* package (version 1.42.0), which applies a fast MCS algorithm tolerant of minor atom/bond mismatches [9].

Two similarity metrics were calculated for each drug pair: the Tanimoto coefficient (based on binary fingerprints) and the structural overlap coefficient (quantifying substructure inclusion) [7,19]. Resulting similarity matrices were clustered using hierarchical clustering with average linkage, with matrix selection guided by cophenetic correlation to preserve pairwise distances. The structural clusters obtained from this analysis served as a reference standard for comparison with those derived from the B-index application, enabling an assessment of the concordance between pharmacogenomic and chemical similarity approaches.

### 2.6. Gene–Drug Bipartite Network Modeling

To visualize pharmacogenomic relationships from the gene–drug correlation analysis, a bipartite network was constructed with two node types —drugs and genes— representing statistically significant interactions filtered as described in Section 2.2 [13]. The network illustrates associations between FDA-approved anticancer drugs and cancer-relevant genes that met defined correlation thresholds, offering an intuitive and scalable platform for exploring subnetworks and generating hypotheses.

Built in Cytoscape (version 3.10.2), the network comprises 124 drugs and 399 protein-coding genes, linked by 1304 significant correlations. A force-directed layout was applied to enhance readability by minimizing edge crossings. Edge width was scaled to reflect the strength of Pearson correlations, highlighting stronger associations. The bipartite structure enforces connections only between genes and drugs, supporting topological analyses such as projecting drug similarity based on shared gene associations.

Drug clusters or specific gene subnetworks were filtered and analyzed using Cytoscape’s filtering and network analysis capabilities to deeply investigate local patterns of interaction within the larger context of pharmacogenomics. The results and networks were allocated in GEDA [13], that is a bioinformatics web server platform available online. This visualization framework provided the foundation for mapping B_index_-derived similarity patterns onto specific drug-gene subnetworks, enabling the identification of functionally related drug clusters as described in subsequent analyses.

## 3. Results

### 3.1. Construction of a Drug-Gene Target Bipartite Network

**Figure 1:**
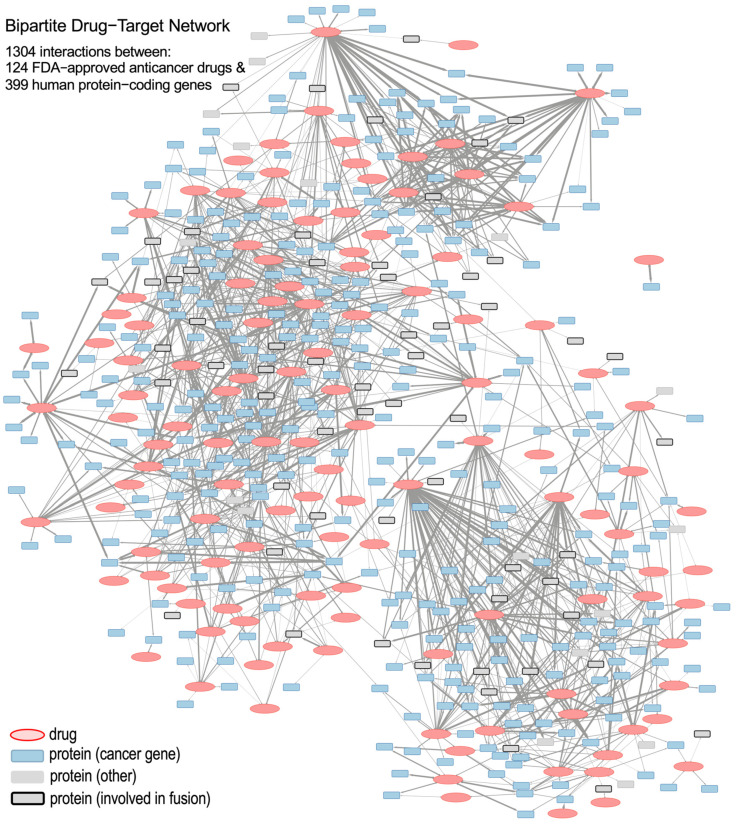
Schematic representation of the bipartite drug-to-gene network, which includes 124 anticancer drugs (red ellipses) and 399 protein-coding genes (blue and gray squares) as nodes; as well as 1304 edges (links) corresponding to statistically significant correlations between drugs and genes. Protein squares that correspond to the genes involved in gene fusion are marked with thick lines.

We constructed a bipartite pharmacogenomic network of FDA-approved anticancer drugs and cancer gene targets from statistically and activity-based filtered gene–drug relationships. The network includes 124 unique anticancer drugs, 399 protein-coding genes, and 1304 statistically significant correlations derived from analyzing a panel of 60 cancer cell lines (NCI-60). Figure 1 shows the network schematically. The complete corresponding data for the bipartite drug-gene target network are provided as a Cytoscape file (see File S2) and an Excel table (see Table S1).

The edges of the bipartite network denote positive and significant correlations that exist between the activity of drugs and the expression of genes. The width of an edge signifies the absolute value of the Pearson correlation coefficient. Drug nodes are colored red, and gene nodes (i.e., drug targets protein-coding genes) are colored according to their functional category. These categories include: cancer genes (either oncogenes or tumor-suppressor genes, TSGs), fusion genes, and other genes (that are not classified in the previous categories). This color coding allows for a quick visual interpretation of the gene subtype and the drug–gene connectivity patterns.

The network topology in Figure 1 reveals a complex architecture with heterogeneous drug-to-gene interaction subnetworks. These subnetworks range from multi-gene–drug hubs to multi-drug–gene interactions. They offer a more specific overview of pharmacogenomic interactions and serve as the basis for studying localized gene–drug modules in our subsequent analysis.

### 3.2. Identification of Known and Putative FDA-Approved Cancer Drug–Gene Interactions

**Figure 2:**
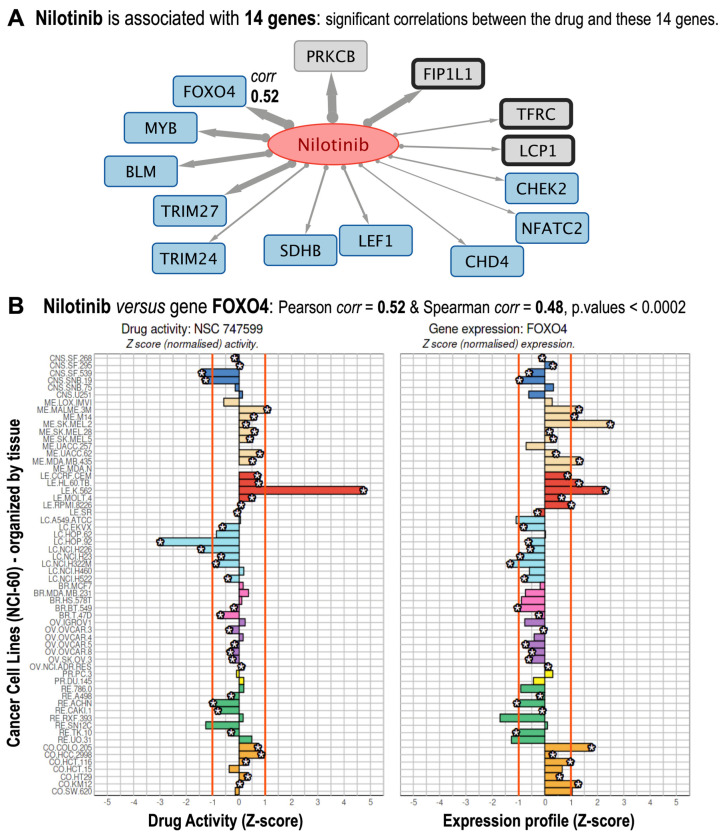
Gene targets found associated with Nilotinib. (**A**) Subnetwork corresponding to the 14 genes that are found significant targets for nilotinib. The links in gray are directed from the drug to the genes, and the thickness of each link is proportional to the correlation. (**B**) Nilotinib versus FOXO4 gene shows correlation values: Pearson corr = 0.52, Spearman corr = 0.48, both significant with Holm-adjusted *p*-values < 0.0002. The figure shows the drug activity profile for nilotinib (NSC 747599) and the gene expression profile for FOXO4. Both are shown as normalized Z-sores in 60 cancer cell lines, which are organized by cancer types affecting different tissues: central nervous system (dark blue); melanoma-skin (pale beige); leukemia-blood (red); lung (light blue); breast (pink); ovarian (purple); prostate (yellow); renal (green); and colon and rectum (pale brown).

To determine clinical relevance, we examined the bipartite network for known and well-reported interactions, as well as new interactions that could lead to novel drug–target associations (Figure 1). As an example of well-stablished interactions, nilotinib, a second-generation BCR-ABL tyrosine kinase inhibitor of Chronic Myeloid Leukemia (CML), was associated with known targets such as BCR-ABL1, KIT, and PDGFR [20]. In fact, 14 genes showed strong correlations with nilotinib (Pearson’s r ≥ 0.334; Holm-adjusted *p* ≤ 0.05), validating known interactions and highlighting novel ones with plausible mechanistic significance (Figure 2A).

For instance, transcription factor FOXO4 (Pearson r = 0.52; Spearman’s correlation = 0.48) controls apoptosis and quiescence through tyrosine kinase signaling pathways [21] (Figure 2B). As a non-canonical nilotinib target, a positive correlation would suggest that nilotinib indirectly controls FOXO4 activity through nilotinib-sensitive pathways.

**Figure 3:**
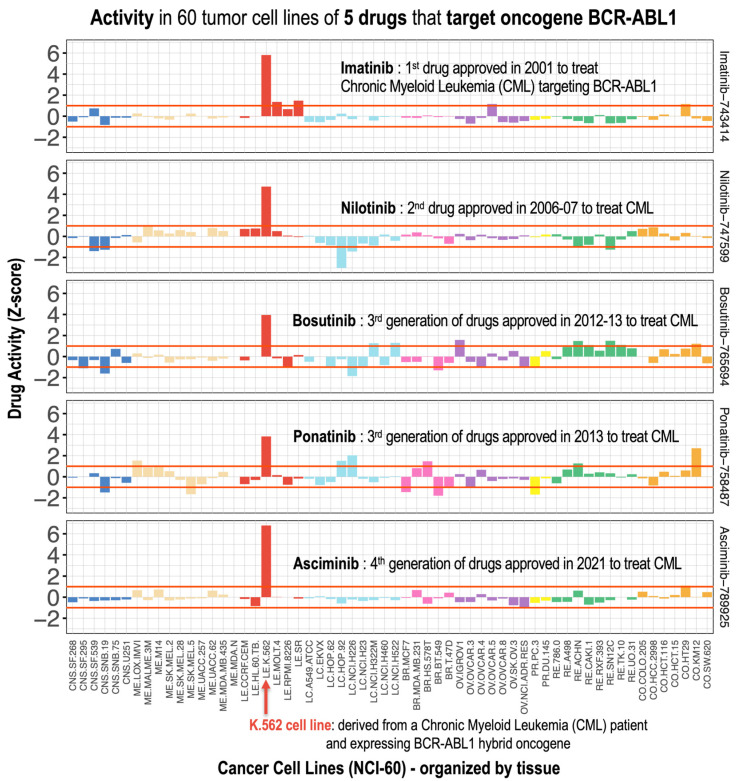
Pharmacological activity profiles of five anticancer drugs: imatinib, nilotinib, bosutinib, ponatinib, and asciminib. For comparison, the profiles of these drugs are presented horizontally (one above the other) in 60 tumor cell lines and with the same normalized Z-score scale on the X-axis. These five drugs correspond to different generations of inhibitors of the chimeric BCR-ABL1 fusion gene, and are therefore used for the treatment of Philadelphia chromosome-positive Chronic Myeloid Leukemia (Ph+ CML). The K.562 cell line shows the greatest activity, as it contains the BCR-ABL1 chimeric oncogene.

Moreover, given the importance of nilotinib as a drug that targets BCR-ABL [22], we investigated its activity profile in comparison to those of imatinib, bosutinib, ponatinib, and asciminib (Figure 3). These five drugs correspond to different generations of inhibitors of the quimeric fusion gene BCR-ABL1, and are all used to treat of Philadelphia chromosome-positive Chronic Myelogenous Leukemia [22]. Validation of NCI-60 activity profiles for the five BCR-ABL1 inhibitors in leukemia versus non-leukemia cell lines revealed differential activity profiles, with K-562 cells being particularly sensitive, which is consistent with prior studies [22].

Importantly, our correlation-based approach captures non-binding interactions that reflect downstream or parallel pathway interactions, broadening the classical definition of drug targeting beyond direct biochemical evidence. These results have implications for biomarker discovery, the development of combination therapies, and the prediction of resistance. Although experimental verification is required, stringent statistical filtering, including double correlation approaches and the Holm *p*-value correction, ensures biological plausibility and low false-positive rates.

### 3.3. Pairwise Drug-to-Drug Clustering Based on the Analysis of the Common Gene Interactions

**Figure 4:**
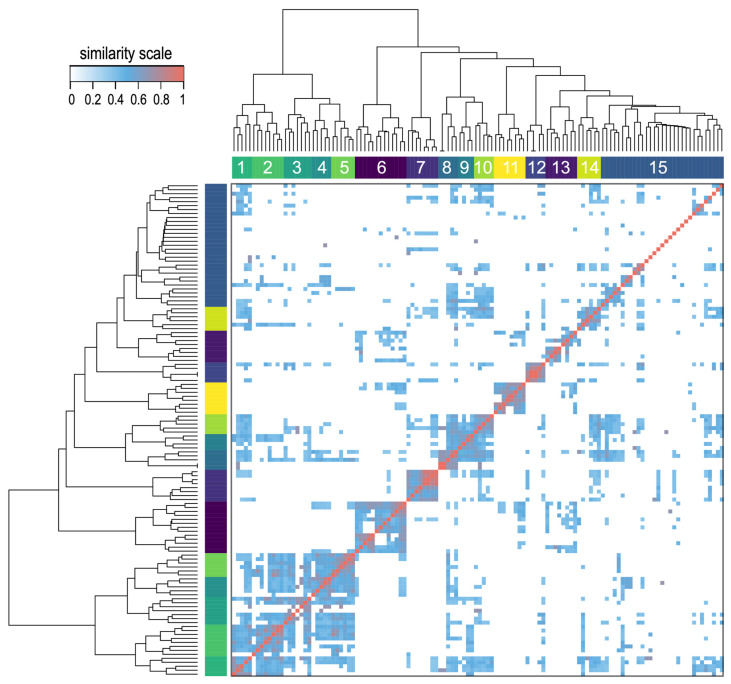
Heatmap obtained using the B similarity index in the pairwise comparison of 124 anticancer drugs. The B-index is derived from the analysis of the potential gene targets that are shared by each drug pair. The heatmap includes a hierarchical clustering of the drugs and the corresponding dendrogram, both based on the pairwise drug-to-drug distance. The clustering analysis provides fifteen main groups numbered from 1 to 15. The similarity scale is included to indicate the color scale of the heatmap.

We calculated all pairwise drug-to-drug similarities using the B-index, which was described in the Materials and Methods section. This index maintains gene target overlaps without severely penalizing dissimilar gene set sizes. Furthermore, we transformed the resulting 124 × 124 pairwise similarity matrix into a distance matrix. We analyzed this matrix using a hierarchical clustering algorithm and represented the results in the form of a heatmap (Figure 4). This analysis revealed 15 strong drug clusters. These clusters were biologically meaningful, grouping drugs with similar mechanisms of action or molecular classes. A dendrogram derived from the clustering analysis was included with the heatmap to illustrate the proximity of different drugs. A detailed view of the dendrogram is shown in Figure S1, including all drug names to facilitate searching over the hierarchical relationships and clusters formed by the drugs. Overall, this analysis effectively grouped mechanistically related compounds (e.g., kinase inhibitors, nucleoside analogs), highlighting the value of the B-index in capturing functional relationships. The results suggest that pharmacogenomic similarity complements structural similarity, with implications for drug repurposing and combination therapy design.

A focused view on the heatmap (Figure 4) and the dendrogram (Figure S1) allowed finding out relevant examples of drug proximity. For example, the two well-known BCR-ABL inhibitors –imatinib and nilotinib– are located together in the same cluster 10, being two drugs that share a common mechanism of action. Similarly, afatinib and neratinib, which are ERBB1 (EGFR) and ERBB2 (HER2) inhibitors, were found together in cluster 11 due to their shared gene targets. They are well-known inhibitors of the ERBB (HER) receptor tyrosine kinase family, i.e., pan-HER inhibitors.

**Figure 5:**
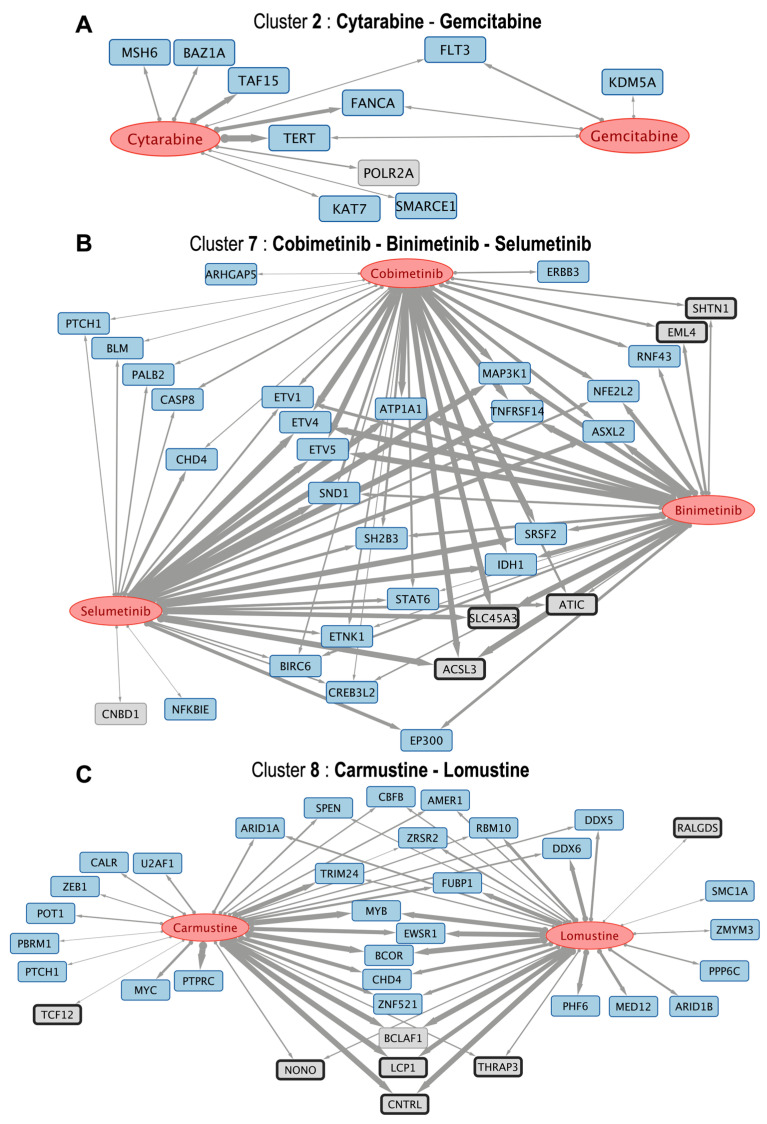
The figure presents three subnetworks of drugs (red ellipses) and their respective gene targets (which show the same color code as in Figure 1). (**A**) cytarabine and gemcitabine (included in cluster 2 in the heatmap) associated with 10 protein-coding genes; (**B**) cobimetinib, binimetinib and selumetinib (included in cluster 7 in the heatmap) associated with 32 genes; and (**C**) carmustine and lomustine (included in cluster 8 in the heatmap) associated with 36 genes. The links have different thicknesses depending on the value of the B-index, which ranges from 0 to 1, indicating similarity and 1 being the maximum.

In a further exploration of the clusters generated in Figure 4 we selected 3 groups of drugs that are close to each other in clustering in order to present and examine the subnetworks of gene targets that they bring together. These drug groups were also examined as subnetworks in Figure 5, that shows drugs with high B-index that clustered together.

The three groups of drugs that clustered together, selected with their associated targets (Figure 5) are: (i) cytarabine and gemcitabine (included in cluster 2); (ii) cobimetinib, binimetinib and selumetinib (included in cluster 7, that are MEK inhibitors); and (iii) carmustine and lomustine (nitrosoureas that are included in cluster 8). The respective drug-to-gene target subnetworks are presented in Figure 5A–C. As an example of an interesting result, the subnetwork of cobimetinib, binimetinib and selumetinib indicates that these drugs can act on genes ETV1, ETV4, and ETV5. These are protein-coding genes that belong to the PEA3 subfamily of ETS transcription factors. These genes have a common DNA-binding domain known as the ETS domain and contribute to diverse cellular processes, including cell proliferation, migration, differentiation, and development. In particular, these genes have been found to be associated with various types of cancer, such as prostate cancer, where ETV1 and ETV4 were often found overexpressed. The effect of these drugs on these transcription factors has not been clearly described until now.

### 3.4. Determination of Drug-to-Drug Structural Similarities

To complement the pharmacogenomic clustering analysis, we used cheminformatics techniques to independently analyze drug structural similarities. We processed drug identifiers (NSC and SMILES) via ChemMine Tools and Open Babel to calculate physicochemical descriptors. We utilized the *fmcsR* algorithm to perform structural comparisons, which calculates the Maximum Common Substructure (MCS) and yields Tanimoto and overlap coefficients for all drug-to-drug pairwise comparisons. These coefficients provide a measure of the structural similarity between each drug pair. This analysis generated a data table containing 15,376 comparisons, which is presented in Table S2.

**Figure 6:**
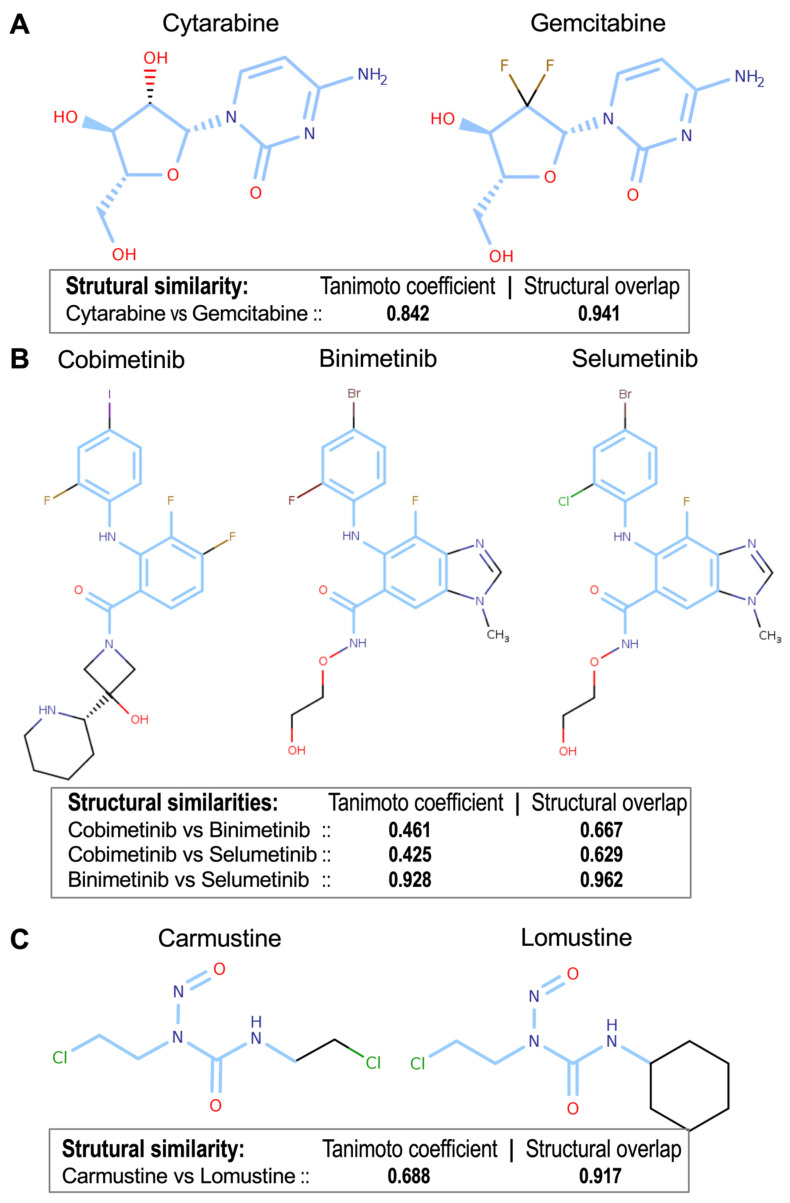
Chemical structures of the three drug groups presented in Figure 5, as well as the structural similarities obtained by pairwise comparison of the drugs using the Tanimoto and the Structural Overlap coefficients. (**A**) cytarabine & gemcitabine (Tanimoto = 0.842, Structural Overlap = 0.941); (**B**) cobimetinib, binimetinib & selumetinib (structural similarity coefficients for the three pairwise comparisons provided); (**C**) carmustine & lomustine (Tanimoto = 0.688, Struct. Overlap = 0.917). The structural analysis reinforces the pharmacogenomic clusters observed in Figure 4. The blue regions in the chemical structures of the compounds indicate the Maximum Common Substructures (MCS).

Following the structural comparison, we used the pairwise structural similarity of the drugs to generate a distance matrix and perform a clustering based on these distances. Hierarchical structural clustering (see Figures S2 and S3) revealed clusters of drugs that significantly overlap with the clusters obtained using the B-index (see Figure S1). Notably, cytarabine and gemcitabine (Tanimoto = 0.842, overlap = 0.941) as well as MEK inhibitors binimetinib and selumetinib (Tanimoto = 0.928, overlap = 0.962) exhibited structural congruence, aligning with their pharmacological similarities (Figure 6A,B). Conversely, intermediate structural similarities, such as those between carmustine and lomustine (Tanimoto = 0.688, overlap = 0.917), revealed different structure-function relationships because they share many common gene targets, but they also have a large proportion of different ones (Figure 6C). These findings demonstrate that structural analysis reinforces pharmacogenomic clusters, and at the same time provide complementary insights for drug classification and potential repurposing strategies.

### 3.5. Global Drug-to-Drug Comparison Using Common Targets and Structural Similarity

**Figure 7:**
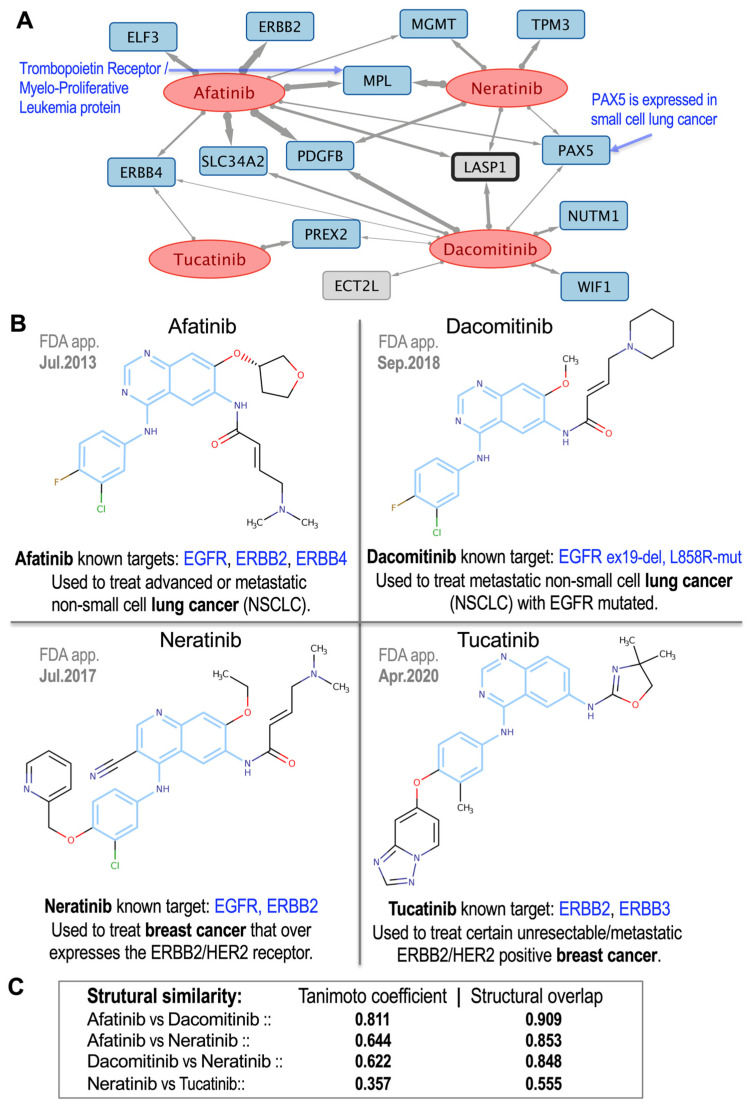
Group of 4 tyrosine kinase inhibitor (TKI) drugs widely used to treat breast and lung cancers: afatinib, dacomitinib, neratinib and tucatinib. (**A**) Pharmacogenomic subnetwork obtained for these 4 drugs, which showed significant association with 14 protein-coding genes. (**B**) Chemical structures of the 4 drugs (blue regions of the compounds correspond to the Maximum Common Substructures, MCS). The year in which each drug was approved by the FDA (FDA app.), the known molecular targets (which are all members of the ERBB family), and the main cancer types for which these drugs are used by oncologists are shown next to each drug. (**C**) Pairwise structural similarity coefficients corresponding to these 4 drugs. The blue regions in the chemical structures of the compounds indicate the MCS.

For a more comprehensive example, we examined a group of commonly used tyrosine kinase inhibitors (TKIs) that are widely used to treat breast and lung cancers: afatinib, dacomitinib, neratinib and tucatinib. The pharmacogenomic network obtained for these four drugs is shown in Figure 7A, and their chemical structures along with their pairwise structural similarity coefficients are presented in Figure 7B,C. The network interactions (Figure 7A) highlighted key molecular links, with afatinib exhibiting a strong correlation with ERBB2, which aligns with its known mechanism in HER2-positive tumors [23]. At the same time, novel strong associations such as afatinib with SLC34A2 or neratinib with MPL (myeloproliferative leukemia protein, proto-oncogene) suggest previously unexplored targets that may be quite relevant to better understand the therapeutic mechanisms of these drugs.

Structural analysis (Figure 7B,C) revealed variable similarities: high concordance between afatinib–dacomitinib (Tanimoto = 0.811; overlap = 0.909), moderate similarity for afatinib–neratinib (Tanimoto = 0.644; overlap = 0.853) and dacomitinib–neratinib (Tanimoto = 0.622; overlap = 0.848), and lower similarity for neratinib–tucatinib (Tanimoto = 0.357; overlap = 0.555), consistent with their distinct selectivity profiles. Overall, our analysis revealed 1304 robust gene–drug associations between 124 anticancer drugs and 399 genes, thereby establishing a comprehensive pharmacogenomic framework. The integration of putative gene targets and chemical structural analysis validates anticancer drugs similarity and uncovers novel mechanistic hypotheses for oncology applications.

### 3.6. Agreement Between Drugs Structural Similarity and Drugs Common Gene Targets

A drug-to-drug structural comparison provides a strong support for understanding the common chemical and biomolecular actions of drugs which have strong structural similarities. This understanding is behind the robust rational that compounds with very similar chemical structures will have a very similar activity to pharmacological agents. Within this framework, we used the structural similarity of the anticancer drugs to determine whether the analysis of their potential gene targets, as identified in our pharmacogenomic analysis, yields similarity values that are concordant with the drugs’ structural similarity. As described above, we measured the drug-to-drug similarity within the bipartite network of drug–target genes (Figure 1), reading the number of genes associated with each drug and calculating for each pair of drugs the putative targets that they have in common (as in the subnetworks shown in Figure 5 and Figure 7A). To be more accurate in the measurement of this overlap, we calculated for each drug pair the classical statistical indexes used to gauge the similarity of two groups (the Jaccard index and the Sørensen–Dice index). Furthermore, as described in Section 2.4 of the Methods, we also designed a novel index (B-index) to calculate such overlap. These three similarity coefficients (B-index, Jaccard and Sørensen–Dice) were calculated for all pairwise comparisons of the 124 anticancer drugs (a total of 7626 comparisons). Only 1337 pairs had at least one common gene target in the network, while 6289 pairs had no intersection. Table S3 provides the complete list of values for the B-, Jaccard- and Sørensen–Dice- indexes corresponding to 1337 drug pairs. To provide some illustrative examples of this analysis, Table 1 presents the information corresponding to 9 drug pairs that were shown in Figure 5A–C and Figure 7B.

## 4. Discussion

### 4.1. Drug–Gene Bipartite Network: Drug Activity and Gene Expression Analysis

As shown in Figure 1, we developed a bipartite network that integrates FDA-approved cancer drugs and human genes expression, providing insights into the potential activity of anticancer drugs against multiple therapeutic targets. Unlike databases like STITCH [24], our method emphasizes statistical correlation between expression profiles and drug sensitivity, identifying biologically relevant interactions. Known interactions (e.g., nilotinib–BCR-ABL1, afatinib–ERBB2) were accurately captured, validating our method. Drug clusters (e.g., cytarabine–gemcitabine, carmustine–lomustine) aligned with clinical mechanisms, further supporting functional validity.

### 4.2. Development of a New Index for the Association Between Drugs

To enhance drug similarity measurement, we developed the B-index, a novel similarity metric based on the number of common elements shared by two features (in our case, two drugs), which preferentially emphasizes similarity by considering the number of elements that each feature has (i.e., the number of genes that each drug presents). In contrast to the Jaccard index, which punishes set size differences [7], the B-index penalizes small overlaps even if absolute numbers of genes are different. Thus, the index has greater sensitivity to drug pharmacogenomic similarities, particularly when there are limited annotations.

The drug clusters thus obtained (Figure 4) were investigated to determine whether the clustering proximity was related to biochemical and functional similarity. Binimetinib, cobimetinib, and selumetinib, all MEK inhibitors, clustered together and shared many targets, such as ETV1, ETV4 and ETV5, which play crucial roles in development, organogenesis, and cell proliferation, and are also implicated in cancer; or MAP3K1 and TNFRSF14 which are involved in the regulation of cell migration, survival, and apoptosis. Furthermore, MAP3K1 can influence JNK signaling, which can be modulated by TNFRSF14 through the activation of the EGFR pathway [25]. This concordance between the similarity score and common biological functions serves to confirm the applicability of the B-index to the analysis of pharmacogenomic data. Although the B-index does not fulfill the triangle inequality, it produces clusters with greater biological relevance than standard metrics in this context. Therefore, it provides an alternative approach to determining drug similarity aside from chemical structure.

### 4.3. Identification of Known and Novel Interactions Between FDA-Approved Drugs and Cancer Genes

The analysis of drug–gene subnetworks yielded strong validation of established interactions and revealed novel ones with high biological plausibility. As we described in the Results section, the drug–gene associations that we proposed (i.e., the network presented in Figure 1) were not established from manually curated databases or binding assays, but through statistically significant correlations between drug sensitivity and gene expression profiles in cancer cell lines. While correlation does not imply causation, the shared occurrence of biologically relevant genes across various drugs lends credence to the interpretive power of this strategy.

For instance, the subnetwork between cytarabine and gemcitabine (Figure 5A) —both nucleoside analogs—shared interactions with TERT, the telomerase catalytic subunit [26]. Since telomerase activation facilitates proliferation and viability in most tumors, this implies that cytarabine and gemcitabine can exhibit anti-tumor activity not only through DNA incorporation but also through targeting telomere maintenance vulnerabilities [27]. This is concordant with their common clinical application in highly proliferative malignancies.

Similarly, as mentioned above, three MEK inhibitors (binimetinib, selumetinib, and cobimetinib) clustered together, showing common correlations with ETV4, ETV5, and TNFRSF14 (Figure 5B). ETV4 and ETV5 of the ETS family are involved in cancer stemness and metabolic reprogramming [28], whereas TNFRSF14 inhibits tumor growth through apoptotic signaling [29]. These associations are coherent with the biological function of MEK inhibition in regulating downstream effectors of the RAS–ERK pathway [30]. Cobimetinib, despite not being structurally as similar (Tanimoto < 0.5), clusters functionally with the others, highlighting the strength of the B-index in identifying shared pharmacogenomic activity even with low structural similarity.

Another case is carmustine and lomustine, two nitrosoureas, which exhibit a large overlap of associated genes, most prominently BCLAF1, an apoptosis-associated transcriptional regulator (Figure 5C) that has been proposed as a potential therapeutic target [31]. This finding is consistent with their established use in brain tumors and supports correlation-based clustering [32].

EGFR TKIs, including dacomitinib and afatinib, are key drugs used to treat Non-Small Cell Lung Cancer (NSCLC). Their therapeutic effects can be significantly improved through their combination with FAK inhibitors, which results in enhanced cell death and decreased tumor growth due to the inhibition of major signaling pathways, such as AKT phosphorylation [33]. Figure 7 shows the positive correlations of these drugs with LASP1, a promising therapeutic target in NSCLC due to its involvement in promoting tumor malignancy via the FAK-AKT pathway [33,34].

These results illustrate the biological interpretability and translational relevance of the correlation-derived network. The B-index optimizes drug–drug similarity based on overlapping gene effects, even between structurally unrelated compounds, providing an avenue for therapeutic repurposing by identifying drugs with similar genomic footprints for use in new cancer settings. The B-index score between lapatinib and bosutinib (B = 1.0), despite target class disparities, indicates functional redundancy or unanticipated overlap in gene regulation.

These observations are particularly valuable in drug repositioning scenarios, where the established safety profiles of previously used drugs facilitate pharmacological translation. The pharmacogenomic correlations identified by the B-index reveal molecular convergence consistent with biological function, mechanistic classification, and therapeutic opportunity. Several novel associations (e.g., nilotinib–FOXO4, MEK inhibitors–ETS family genes) highlight potential biomarkers and mechanistic hypotheses. Together, these findings support the use of network-based drug profiling as a tool for developing actionable drugs, creating personalized treatment strategies, and making clinically relevant inferences that warrant experimental validation.

### 4.4. Structural Similarity Between Drugs and Shared Gene-Network: The Case of the ERBB Family

Structural homology among anticancer agents is often accompanied by functional homology, including common gene targets and clinical applications. Structurally similar compounds tend to regulate the same biological pathways, a phenomenon supported by our network analyses (Figure 1, Figure 4 and Figure S1), where chemical analogs shared gene associations [19,35].

Afatinib, for example, is linked to ERBB2 (HER2), a receptor tyrosine kinase overexpressed in numerous cancers (Figure 7). Its overexpression is associated with poor prognosis and increased tumor growth, notably in breast and thyroid cancers [36]. Afatinib inhibition of ERBB2 interrupts these oncogenic signals, consistent with its therapeutic activity against HER2-driven tumors [37]. Both afatinib and dacomitinib were also linked with SLC34A2, a sodium-phosphate transporter that plays a role in tumorigenesis. SLC34A2 overexpression promotes cell proliferation via c-MYC regulation and ALK pathway stabilization [38,39]. Since afatinib does not directly inhibit SLC34A2, this association suggests that it may indirectly modulate the transporter through upstream pathways, indicating the existence of other potential targets for further investigation of afatinib’s therapeutic use.

Afatinib and neratinib both target EGFR (ERBB1) and HER2 (ERBB2), but with different specificity profiles. Afatinib is an irreversible EGFR and HER2 inhibitor with therapeutic activity in HER2-mutated tumors, co-dependent on EGFR activation [37]. Neratinib is a pan-HER kinase inhibitor with preferential activity against HER2-positive breast cancer [40]. Their high structural similarity (0.853) and moderate Tanimoto similarity (0.644) are consistent with these functional overlaps despite differing clinical utility.

Tucatinib is a selective HER2 inhibitor that is both structurally and mechanistically distinct from neratinib. Tucatinib exhibits clinical activity against trastuzumab-resistant HER2+ breast cancer, particularly in cases of brain metastasis [41,42]. Unlike neratinib, tucatinib bypasses pan-HER inhibition, providing an improved safety profile with fewer off-target effects [43]. It is also known to target ERBB3 and, as shown in Figure 7B, appears to target ERBB4 as well. These distinctions are reflected in their lower structural similarity (Tanimoto = 0.357; overlap = 0.555), which highlights the fact that drugs acting on the same receptor class can differ substantially in terms of selectivity and downstream gene interactions.

These results demonstrate that the network of drugs and potential gene targets presented here, together with the B-index developed here that emphasizes genes common to drug pairs and structural similarity measures, often converge to identify functionally related drug pairs. However, discordant pairs (e.g., neratinib–tucatinib) suggest that chemical similarity does not necessarily imply functional similarity. Therefore, combining pharmacogenomic and structural insights enhances our understanding of drug mechanisms, selectivity, and repurposing potential.

### 4.5. Strengths and Limitations of the Proposed Approach, and Comparison with Similar Studies

This study presents a network-based approach for linking FDA-approved cancer drugs with potential gene targets, enabling the consideration of new molecular candidates for targeted cancer treatment. By integrating NCI-60 pharmacogenomic data with annotated COSMIC and DrugBank entries, our dataset provides a detailed biological rationale for each suggested drug–gene association. The B-index, designed to improve upon conventional similarity measures (such as the Jaccard index), captures meaningful overlaps in small or asymmetric gene sets. Cross-validation with structural similarity measures (such as the Tanimoto coefficient) also lends support to the robustness of the identified drug clusters.

While the B-index is particularly effective when comparing drugs with differently sized target sets, caution is warranted when both drugs have very few annotated targets, since even a single shared gene can disproportionately raise the similarity score. In such cases, complementary evidence from structural similarity or biochemical studies should be considered.

It is also important to recognize that all identified associations are correlational and may reflect broader regulatory relationships rather than direct drug–target interactions. Additionally, the NCI-60 cell line pharmacogenomic panel is unable to capture features of the tumor microenvironment, such as heterogeneity, immune infiltration, or patient-specific mutations, which limits the translational scope of the findings. Finally, because the analyses are in silico, repurposing candidates require confirmation through in vitro and in vivo experimental studies. These studies are necessary to verify whether a specific drug can bind to or interact with potential protein targets, and all of this would be needed before clinical application.

Preliminary research initiatives using drug activity in cancer cell lines started long ago with a pioneering study reported in 1989, that performed analysis of patterns of differential activity of drugs against human tumor cell lines [44]. Later, gene expression profiling was integrated with drug chemoactivity in cancer cell lines [45]. As described in the Introduction, Reinhold and collaborators produced a systematic evaluation of FDA-approved anticancer drugs using pharmacogenomic data. This evaluation provided information on drug sensitivity and genome-wide expression profiles across 60 NCI cancer cell lines, and it was published the resource CellMiner [6,11,46]. More recently, machine-learning approaches using NCI-60 data have been applied for gene target prediction and drug similarity analysis [47], as well as to predict multi-relational drug-gene interactions and detect novel off-targets [48]. Other initiatives have been undertaken to test the possibility of repurposing drugs for cancer treatment through in silico molecular target prediction [49]. In the last year, artificial intelligent (AI) models have been applied for virtual screening of large compound libraries on cancer cell lines as a novel approach for drug discovery [50]. While all of these research strategies share some similarities with the methodology used in the present work, our specific analysis and results, which provide many potential gene targets for 124 anticancer drugs, are novel and complementary to the current efforts for molecular target prediction. Finally, there are several relevant drug–gene interaction databases that provide networks of associations between drugs and gene targets, which have been obtained using different approaches than the methodology presented here. One of these data resources, currently active, is the Drug–Gene Interaction Database (DGIdb, https://dgidb.org), a publicly accessible resource that aggregates genes, gene products, drugs and drug–gene interaction records [51]. Another is the Cancer Driver Drug Interaction Explorer (CADDIE, https://exbio.wzw.tum.de/caddie/), a web server that integrates human gene–gene and drug–gene interaction databases, with information regarding cancer-related genes, gene expression, and anticancer drugs [52].

### 4.6. Future Perspectives

The network-based strategy described in this study can contribute to rational oncology drug development by revealing functional similarities between drugs that are not evident from structural comparisons alone. Such insights may inform the design of combination therapies that target convergent pathways and aid in predicting cross-resistance across different drug classes. The discovery of novel drug–gene associations also creates opportunities for biomarker development, since correlated expression patterns may indicate predictive markers of therapeutic response. Although this work focused on oncology, the B-index framework could be extended to other therapeutic areas with available pharmacogenomic data, broadening its potential for repurposing and mechanistic exploration. Ultimately, coupling this computational approach with experimental and clinical validation will be essential to accelerate the translation of pharmacogenomic findings into patient-tailored therapies.

## 5. Conclusions

Our network analysis demonstrates the utility of bipartite networks in mapping complex relationships between cancer genes and drugs, as well as in identifying plausible drug targets. The method allows researchers to examine patterns of drug activity on individual cancer genes, providing insights into novel treatment mechanisms.

The innovation in the B-index addresses the drawbacks of the structural similarity coefficient by proposing the identification of similar drugs based on their shared gene networks. The method expedites the discovery of existing and prospective targets, shedding light on the effects of poly-pharmacy and co-therapy in current molecular precision medicine. The B-index offers an enhanced drug similarity metric with broad applicability to identify valid candidates for repositioning or combination therapies.

By explaining known and novel drug–gene interactions through a network-based approach, this study is relevant to the field of precision oncology. It provides opportunities to translate in silico results into clinically testable hypotheses. Our results recapitulate existing drug–drug relationships and reveal novel target associations, confirming the utility of transcriptomics in guiding novel molecular cancer therapies and drug repositioning strategies. Future efforts should focus on experimental validation of predicted drug–gene interactions and clinical validation of repositioning candidates identified using this framework.

## Figures and Tables

**Table 1:** Pairs of anticancer drugs shown with their B-index and Jaccard index, which are calculated based on the number of gene targets they share, and their drug structural similarity coefficients: Tanimoto and MSC score. The drug pairs correspond to those presented in Figure 5 and Figure 7B.

Drug Pair	B-Index	Jaccard Index	Tanimoto Coefficient	Structural Overlap (MCS Score)	Shared Targets	Drug Class
Cytarabine—Gemcitabine	0.542	0.300	0.842	0.941	3	Nucleoside analogs
Cobimetinib—Binimetinib	0.857	0.733	0.461	0.667	22	MEK inhibitors
Cobimetinib—Selumetinib	0.858	0.750	0.425	0.629	23	MEK inhibitors
Binimetinib—Selumetinib	0.805	0.667	0.928	0.962	19	MEK inhibitors
Carmustine—Lomustine	0.715	0.556	0.688	0.917	20	Nitrosoureas
Afatinib—Dacomitinib	0.555	0.385	0.811	0.909	5	TKIs ^1^
Afatinib—Neratinib	0.694	0.500	0.644	0.853	5	TKIs ^1^
Dacomitinib—Neratinib	0.417	0.250	0.622	0.848	3	TKIs ^1^
Neratinib—Tucatinib	0.000	0.000	0.357	0.555	0	TKIs ^1^

^1^ TKIs: Tyrosine Kinase Inhibitors.

## Data Availability

All the research data produced in this study are provided as Supplementary Materials available within the MDPI journal website: https://www.mdpi.com/article/10.3390/pharmaceutics17111421/s1.

## Supplementary Materials

The following supporting information can be downloaded at: https://www.mdpi.com/article/10.3390/pharmaceutics17111421/s1, as Supplementary information and data provided with this article. File S1: Mathematical foundations and proofs for the B-index; File S2: Cytoscape file (.cys) containing all the information from the bipartite drug-gene network (presented in Figure 1) containing 124 anticancer drugs and 399 protein-coding genes, as nodes, and 1304 statistically significant correlations, as links; Table S1: Excel file containing the information from the same bipartite drug–gene network in table format (it is equivalent to the information included in the Cytoscape file, Supp.Mat.S2); Figure S1: Dendrogram corresponding to the hierarchical clustering of 124 anticancer drugs obtained using the B-index (it includes all drug names to facilitate searching, and it is the same dendrogram provided with the heatmap in Figure 4); Table S2: Excel file containing information on the structural comparison of 124 anticancer drugs, showing the Tanimoto coefficients (for the pairwise comparison of the drug chemical structures), and the Structural Overlap coefficients (which is calculated using the Maximum Common Substructure, MCS). The file includes a data table with 7750 drug-to-drug pairwise comparisons. The number of atoms in each drug is indicated as Query_Size (for drug1) and Target_Size (for drug2), and the atoms in common between each pair are indicated as MSC_Size (because the MSC score calculates the structural atomic overlap between each pair of drugs); Table S3: Excel file containing the complete list of values for the B-index, Jaccard-index and Sørensen–Dice-index corresponding to the 1337 drug pairs that share at least one potential gene target in the bipartite drug–gene network shown in Figure 1; Figure S2: Dendrogram corresponding to the hierarchical clustering of 124 anticancer drugs obtained using the Tanimoto coefficients (includes drug names for easier searching); Figure S3: Dendrogram corresponding to the hierarchical clustering of 124 anticancer drugs obtained using the Structural Overlap coefficients.
