# Supplementary material for: Pharmacogenomic Drug–Target Network Analysis Reveals Similarity Profiles Among FDA–Approved Cancer Drugs
Source: Pharmaceutics. 2025 Nov 3;17(11):1421. doi: 10.3390/pharmaceutics17111421 (PMC12655765; doi:10.3390/pharmaceutics17111421)
Supplement: Supplementary file 1 [file pharmaceutics-17-01421-s001.zip › Supplementary_Figures_S1-S2-S3.pdf]

Supplementary Figure S1

Dendrogram: **clustering** based on a **pairwise drug-to-drug distance** (presenting **15 groups**)

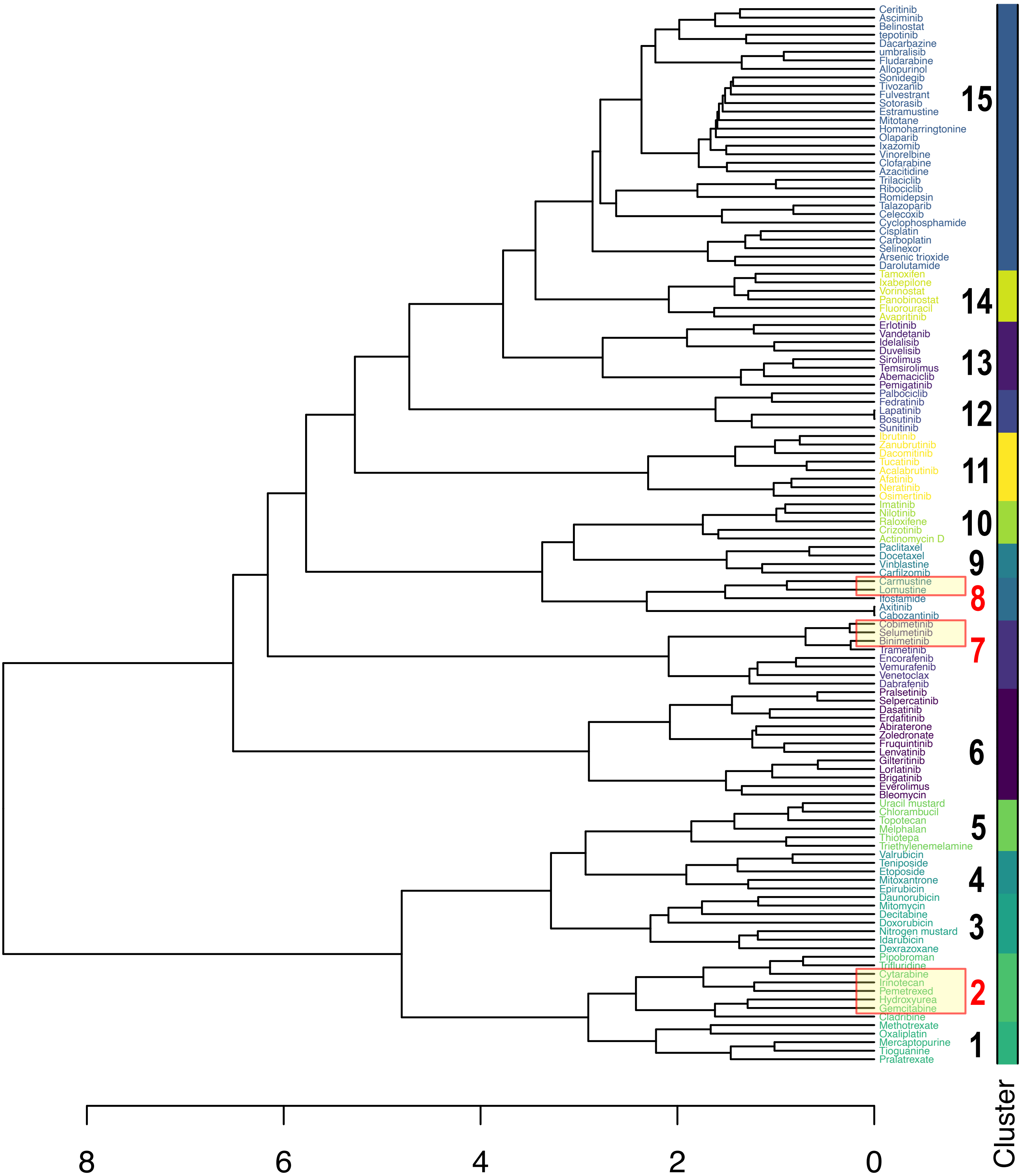

Clustering of **Anticancer Drugs** obtained based on the **distance** derived from the **similarity B-index proposed** (obtained from the **number of common gene targets** that each pair of drugs share). This clustering generates **groups of drugs** with significant **structural similarity**.

Cluster Examples:

Cluster 2	Cluster 7	Cluster 8
**Cytarabine**	**Binimetinib**	**Carmustine**
**Gemcitabine**	**Cobimetinib**	**Lomustine**
	**Selumetinib**	

Supplementary Figure S2

Drugs comparison by **Structural Similarity**, based on chemical ***Tanimoto* coefficient**

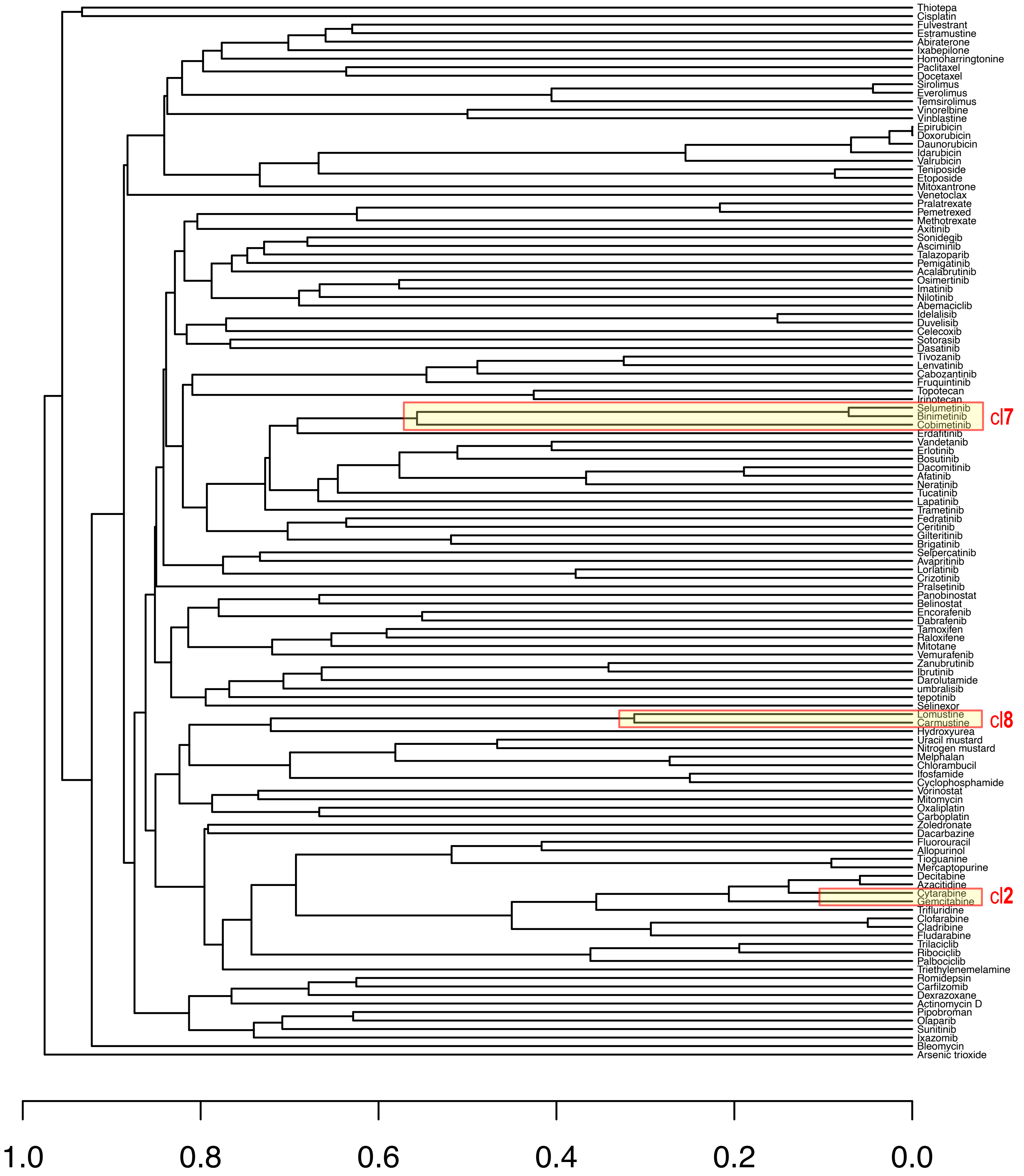

Clustering of **Anticancer Drugs** obtained based on the pairwise **distances** derived from the ***Tanimoto*** coefficient. *Cluster Examples*:

Cluster 2	Cluster 7	Cluster 8
**Cytarabine**	**Binimetinib**	**Carmustine**
**Gemcitabine**	**Cobimetinib**	**Lomustine**
	**Selumetinib**	

Supplementary Figure S3

Drugs comparison by **Structural Similarity**, based on the ***Maximum Common Substructure*** (**MCS**)

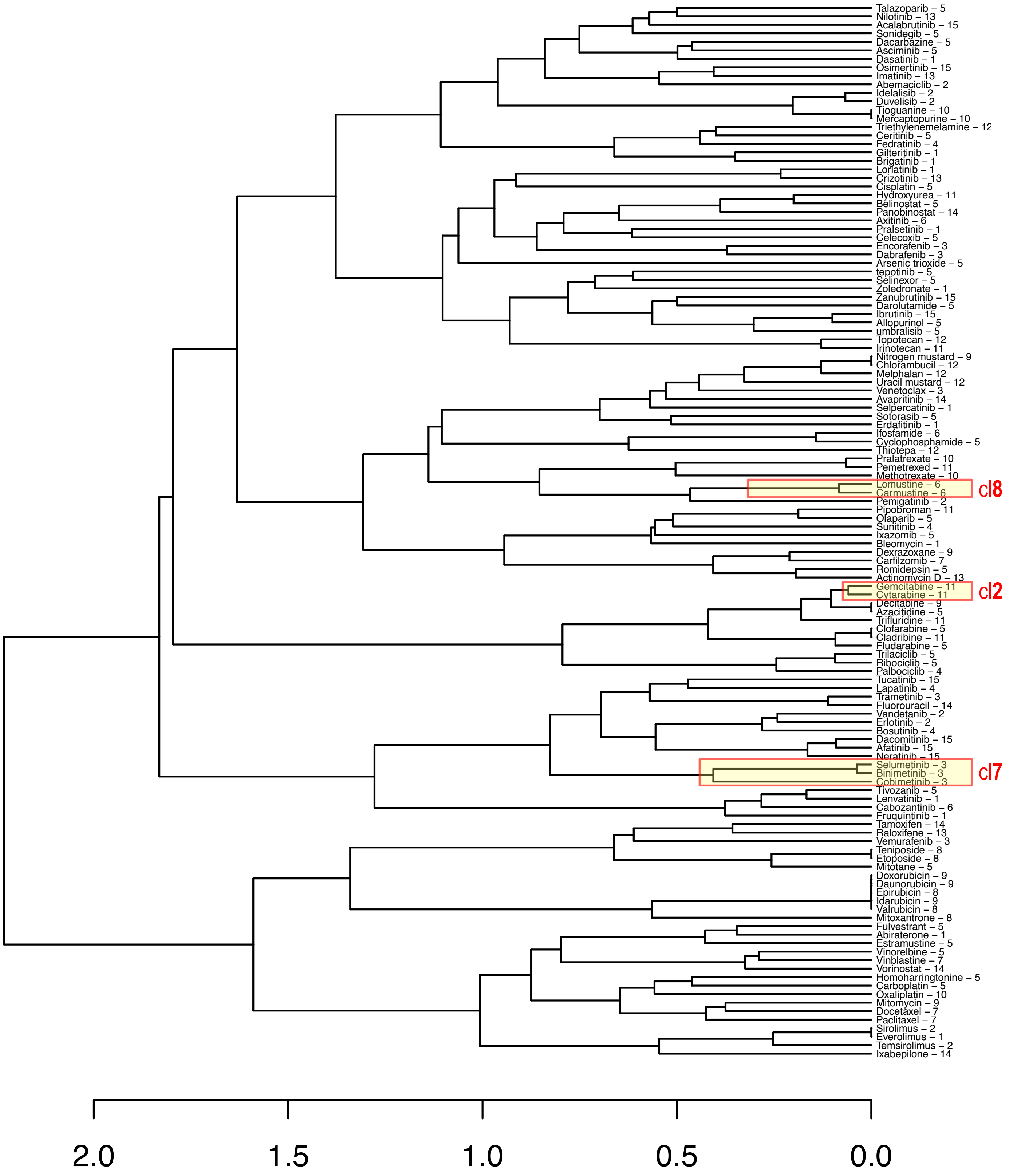

Clustering of **Anticancer Drugs** obtained based on the pairwise **distances** derived from the ***structural overlap*** calculated using the ***MSC***. *Cluster Examples*:

Cluster 2	Cluster 7	Cluster 8
**Cytarabine**	**Binimetinib**	**Carmustine**
**Gemcitabine**	**Cobimetinib**	**Lomustine**
	**Selumetinib**	
