# Supplementary material for: Pharmacogenomic Drug–Target Network Analysis Reveals Similarity Profiles Among FDA–Approved Cancer Drugs
Source: Pharmaceutics. 2025 Nov 3;17(11):1421. doi: 10.3390/pharmaceutics17111421 (PMC12655765; doi:10.3390/pharmaceutics17111421)
Supplement: Supplementary file 1 [file pharmaceutics-17-01421-s001.zip › Supplementary_File_S1_mathematical-proofs-for-the-B-index.pdf]

# Supplementary Material S1

## Mathematical foundations and proofs for the B-index (Berral index)

### S1.1. Formal definition of similarity functions

A similarity function provides a real value that quantifies the closeness between two objects. Let X be a set, a function s: X × X → ℝ is called a similarity on X if s is non-negative, symmetric s(x,y) = s(y,x) and s(x,y) ≤ s(x,x) holds for all x,y ∈ X, with equality if and only if x=y [1].

### S1.2. Existing similarity coefficients

There exist many similarity coefficients where x and y are sets, such as the widely used Jaccard (or Tanimoto) index [2,3], the Russell-Rao [4], and the Sørensen-Dice coefficient [5,6]. These three indices vary between 0 (no shared elements) and 1 (all elements shared), each useful depending on the focus of interest.

Given two sets x, y ≠ ∅, the definitions are:

$$\textit{Russell-Rao}(x,y) = RR(x,y) = \frac{|x \cap y|}{|x| \cdot |y|}$$
$$Jaccard(x,y) = J(x,y) \; \frac{|x \cap y|}{|x \cup y|} = \frac{|x \cap y|}{|x| + |y| - |x \cap y|}$$
$$\textit{Sørensen-Dice}(x,y) = SD(x,y) = \frac{2\,|x \cap y|}{|x| + |y|}$$

### S1.3. Motivation for the Berral index

In our pharmacogenomic data, we have sets with widely varying sizes, and traditional similarity coefficients are adversely affected when comparing sets of different sizes. We aimed to find pairs of sets with the most elements in common, requiring a similarity index that grows with the cardinality of their intersection but is not penalized by the cardinality of their union. When one set is extensive, the union set is also large, which unfairly penalizes traditional measures.

We find it particularly relevant when a small gene set shares elements with another set, even if the latter is large. Therefore, we developed an index that decreases with the size of each individual set rather than the union set, taking into account the impact of intersection size on each set separately.

### S1.4. Formal proof that the Berral index is a similarity function

**Proof that B(x,y) is a similarity:**

1. **Non-negativity:** B(x, y) ≥ 0, as it is a sum of positive terms.
2. **Symmetry:** B(x, y) = B(y, x), by the commutative property of intersection and addition.
3. **Reflexivity condition:** B(x, y) ≤ B(x, x) for all x,y, and B(x, y) = B(x, x) if and only if y = x.

a. $B(x, y) \leq 1$ and $B(x, x) = 1$

b. $B(x, y) = B(x, x) \Leftrightarrow \frac{1}{2} \cdot |x \cap y| \cdot \left(\frac{1}{|x|} + \frac{1}{|y|}\right) = 1 \Leftrightarrow$

$|x \cap y| = |x|$ and $|x \cap y| = |y| \Leftrightarrow$

$x \cap y = x$ and $x \cap y = y \Leftrightarrow$

$x \subseteq y$ and $y \subseteq x \Leftrightarrow x = y$

## S1.5. Properties of the Berral index

For three non-empty sets x, y, z, the Berral index fulfills the following properties:

i) $0 \leq B(x,y) \leq 1$

ii) If $x \subset y \Rightarrow B(x,y) = \frac{1}{2} \cdot \left(1 + \frac{|x|}{|y|}\right)$

iii) If $|x \cap y| = |x \cap z|$, then $|z| > |y| \Rightarrow B(x,z) < B(x,y)$

In the particular case where $x \subset y$ *and* $x \subset z$, $|z| > |y| \Rightarrow B(x,z) < B(x,y)$

iv) $\lim_{|y| \to \infty} B(x,y) = \frac{1}{2} \cdot \frac{|x \cap y|}{|x|}$

Property (iv) indicates that the Berral index gives greater importance to common elements between two sets when one set is not large, particularly when one set is smaller than the other, since shared elements are more improbable in small sets.

## S1.6. Ordering relationship proof

**Theorem:** For any sets x, y ≠ ∅, the indices are ordered as: RR(x,y) ≤ J(x,y) ≤ SD(x,y) ≤ B(x,y)

**Proof:**

**Case 1:** If $x \cap y = \emptyset \Longrightarrow |x \cap y| = 0 \Longrightarrow RR(x,y) = J(x,y) = SD(x,y) = B(x,y) = 0$

**Case 2:** If $x \cap y \neq \emptyset \Longrightarrow |x \cap y| \geq 1$

a. $RR(x,y) \leq J(x,y) \Leftrightarrow \frac{|x \cap y|}{|x| \cdot |y|} \leq \frac{|x \cap y|}{|x| + |y| - |x \cap y|} \Leftrightarrow |x| + |y| - |x \cap y| \leq |x| \cdot |y|$

$\Leftrightarrow |x| - |x \cap y| \leq |x| \cdot |y| - |y| \Leftrightarrow |x| - |x \cap y| \leq |y| \cdot (|x| - 1) \Leftrightarrow \frac{|x| - |x \cap y|}{|x| - 1} \leq |y|$

And this inequality holds, since

$|x \cap y| \geq 1 \Rightarrow |x| - |x \cap y| \leq |x| - 1 \Rightarrow \frac{|x| - |x \cap y|}{|x| - 1} \leq 1$ and $1 \leq |y|$ as $y \neq \emptyset$

b. $J(x,y) \leq SD(x,y) \Leftrightarrow \frac{|x \cap y|}{|x| + |y| - |x \cap y|} \leq \frac{2\,|x \cap y|}{|x| + |y|} \Leftrightarrow |x| + |y| \leq 2\,(|x| + |y| - |x \cap y|)$

$\Leftrightarrow 0 \leq |x| + |y| - 2\,|x \cap y| \Leftrightarrow 2\,|x \cap y| \leq |x| + |y|$

This inequality holds since

$|x \cap y| \leq |x|$ and $|x \cap y| \leq |y|$

c. $SD(x,y) \leq B(x,y) \Leftrightarrow \frac{|x \cap y|}{|x| + |y|} \leq \frac{1}{2} \cdot |x \cap y| \cdot \left(\frac{1}{|x|} + \frac{1}{|y|}\right) \Leftrightarrow \frac{4}{|x| + |y|} \leq \frac{1}{|x|} + \frac{1}{|y|}$

$\Leftrightarrow \frac{4}{|x| + |y|} \leq \frac{|x| + |y|}{|x| \cdot |y|} \Leftrightarrow 4\,|x|\,|y| \leq (|x| + |y|)^2$
$\Leftrightarrow 4\,|x|\,|y| \leq |x|^2 + |y|^2 + 2\,|x|\,|y| \quad \Leftrightarrow 0 \leq |x|^2 + |y|^2 - 2\,|x|\,|y|$
$\Leftrightarrow 0 \leq (|x| - |y|)^2$
Which always holds

## S1.7. Illustrative examples

**Example 1:** Consider sets with different cardinalities:

$$x = \{g1, g2, g20\}$$
$$y = \{g1, g2, g30, g40, g50, g60\}$$
$$z = \{g1, g2, g3, g4, g5, g6, g7, g8, g9, g10, g11, g12, g13, g14, g15, g16, g17, g18\}$$

verifying $x \cap y = x \cap z = y \cap z = \{g1, g2\}$, $|y| = 2|x|$, $|z| = 3|y| = 6|x|$.

With $|x \cap y| = |x \cap z| = |y \cap z| = 2$:

| | | |
|---|---|---|
| $RR(x, y) = 0.11$ | $RR(x, z) = 0.04$ | $RR(y, z) = 0.02$ |
| $J(x, y) = 0.29$ | $J(x, z) = 0.11$ | $J(y, z) = 0.10$ |
| $\mathrm{SD}(x, y) = 0.44$ | $\mathrm{SD}(x, z) = 0.19$ | $\mathrm{SD}(y, z) = 0.17$ |
| $B(x, y) = 0.50$ | $B(x, z) = 0.39$ | $B(y, z) = 0.22$ |

**Example 2:** Subset relationships:

$$x = \{g1, g2, g20\}$$
$$y = \{g1, g2, g30, g40, g50, g60\}$$
$$z = \{g1, g2, g3, g4, g5, g6, g7, g8, g9, g10, g11, g12, g13, g14, g15, g16, g17, g18\}$$

verifying $x \cap y = x \cap z = y \cap z = \{g1, g2\}$, $|y| = 2|x|$, $|z| = 3|y| = 6|x|$.

$$RR(x, z) = 0.06 < RR(x, y) = 0.25$$
$$J(x, z) = 0.13 < J(x, y) = 0.50$$
$$SD(x, z) = 0.22 < SD(x, y) = 0.67$$
$$B(x, z) = 0.56 < B(x, y) = 0.75$$

All indices penalize the greater size of z, but the Berral index is the only measure showing >50% similarity for x and z, which seems more realistic when dealing with gene target sets where finding any shared elements in small sets is statistically significant.

### S1.8. Biological interpretation

The Berral index places greater emphasis on identifying small sets of genes that share many commonalities, which is particularly relevant in pharmacogenomic studies, where drugs often target small, specific gene sets. When comparing drug target profiles, the discovery that two drugs share targets becomes more meaningful when one or both drugs have small target sets, as this represents a more specific and potentially therapeutically relevant relationship than would be captured by traditional similarity measures.

## References

1. Todeschini, R.; Consonni, V.; Xiang, H.; Holliday, J.; Buscema, M.; Willett, P. Similarity Coefficients for Binary Chemoinformatics Data: Overview and Extended Comparison Using Simulated and Real Data Sets. *J Chem Inf Model* **2012**, 52, 2884–2901.

2. Jaccard, P. The Distribution Of The Flora In The Alpine Zone.1 *New Phytologist* **1912**, 11(2), 37–50.

3. Tanimoto, T. T. An Elementary Mathematical Theory of Classification and Prediction. **1958**, 11 pages, Publisher: International Business Machines Corporation, New York.

4. Russell, P. F.; Rao T. R. On Habitat and Association of Species of Anopheline Larvae in South-eastern Madras. *Journal of the Malaria institute of India* **1940**, 3(1), 153-178.

5. Dice, L.R. Measures of the Amount of Ecologic Association between Species. *Ecology* **1945**, 26, 297–302.

6. Sorensen, T. A Method of Establishing Groups of Equal Amplitude in Plant Sociology Based on Similarity of Species and Its Application to Analyses of the Vegetation on Danish Commons. *Kongelige Danske Videnskabernes Selskab* **1948**, 5, 1-34.
